# The Effect of General Bone Mineral Density on the Quantity and Quality of the Edentulous Mandible: A Cross-Sectional Clinical Study

**DOI:** 10.3390/dj11010017

**Published:** 2023-01-03

**Authors:** Anda Slaidina, Baiba Springe, Andris Abeltins, Sergio E. Uribe, Aivars Lejnieks

**Affiliations:** 1 Department of Prosthodontics, Riga Stradins University, LV-1083 Riga, Latvia; 2 Department of Orthodontics, Riga Stradins University, LV-1083 Riga, Latvia; 3 Department of Conservative Dentistry and Oral Health, LV-1083 Riga, Latvia; 4 Baltic Biomaterials Centre of Excellence, Headquarters at Riga Technical University, LV-1083 Riga, Latvia; 5 School of Dentistry, Universidad Austral de Chile, Valdivia 5110566, Chile; 6 Clinics “Gailezers”, Riga East Clinical University Hospital, LV-1079 Riga, Latvia; 7 Department of Internal Diseases, Riga Stradins University, LV-1083 Riga, Latvia

**Keywords:** osteoporosis, bone mineral density, jawbone, edentulous mandible, cone beam computed tomography, cortical bone

## Abstract

Background: Osteoporosis is a disease which is characterized by a decrease in general bone mineral density (BMD), resulting in decreased bone strength and an increased risk of bone fractures. The effect of reduced BMD on the jawbones is still not fully understood. The purpose of the study was to evaluate the impact of BMD on the quality and quantity of the edentulous mandible. Methods: The present study included 127 edentulous postmenopausal women who underwent cone beam computed tomography (CBCT) examinations. BMD measurements of the lumbar spine and femoral necks were performed by dual-energy X-ray absorptiometry. In the cross-sectional CBCT images, three different areas of the mandible (lateral incisor, first premolar, and first molar) were selected. The complete mandibular, trabecular, and cortical bone volumes were measured. All measurements were performed on the total mandibular area, and the basal and alveolar parts of the mandible. Results: The volume of the cortical bone was reduced for females with reduced BMD in the lateral incisor and first premolar regions, both in the total mandibular area and in the basal part of the mandible. The trabecular bone volume statistically significantly increased when the BMD decreased in the complete mandibular area and the basal part of the mandible (linear regression). The total bone volume significantly decreased with a decrease in BMD in the basal part of the mandible. Conclusions: Reduced BMD has a negative effect on the quantity and quality of bone in the basal part of the edentulous mandible.

## 1. Introduction

The loss of teeth results in residual ridge resorption (RRR), a chronic, progressive, and permanent process [1]. After tooth loss, residual ridge resorption occurs similarly in all patients, although individual variations between patients are observed [2,3]. RRR is an important factor influencing the prosthetic rehabilitation of edentulous patients. It is very difficult for the dentist to make complete dentures with optimal retention and stability. Moreover, it is more difficult for the patient to adapt to the new dentures. Using dental implants, it is possible to significantly improve the stability and retention of complete dentures, thus also improving the quality of life of patients. One of the most common problems with the insertion of dental implants is the insufficient quantity and quality of alveolar bone [4,5]. The ability to ensure the stability of the primary implant during surgery is important for the successful osteointegration of dental implants [6]. This is a mechanical phenomenon determined by the quality and quantity of local bone [7], the surgical technique [8], and the implant’s design [9]. The most important indicators of bone quality that directly affect the primary stability of an implant are the amount of cortical bone [10,11] and bone density [12]. The amount of cortical bone in different areas of the jawbone is largely determined by anatomical factors [4]. However, large individual variations have been observed, suggesting that other factors, including metabolic factors, affect the mass of cortical bone. Postmenopausal women have a thinner cortical layer than premenopausal women, suggesting that the rapid reduction in estrogen level and subsequent osteoporosis affect bone quality [13].

Like cortical bone, the rate of RRR after tooth loss is determined by a combination of several factors [1]. Metabolic factors, including osteoporosis, are thought to play a key role in RRR [2,14].

Osteoporosis is a skeletal disease characterized by low bone mineral density and microarchitectural deterioration, resulting in increased bone fragility and a significantly higher risk of bone fractures [15,16]. It is a widespread disease in habitats with a temperate climate around the world and is directly linked to age [15]. According to the World Health Organization (WHO), osteoporosis is the second most common disease after cardiovascular diseases [17], and fractures are found in every third woman and every fifth man over 50 years of age [18]. Although the disease is found in both women and men, the most common form is postmenopausal osteoporosis (90% of cases) [19].

The results of clinical studies on how osteoporosis affects the RRR of the edentulous mandible are controversial [20,21,22,23,24,25,26,27]. In addition, most researchers have evaluated bone resorption in two-dimensional examinations [22,23,24,25], which do not provide information about the bones’ width in the buccolingual dimension. Some studies using three-dimensional examinations have found that osteoporosis affects mandibular RRR [20,26], but some studies have not found such a relationship [21]. Studies often have small sample sizes [26], include patients with a wide age range [20], and use methods that are not the gold standard for the diagnosis of osteoporosis [20].

Similarly, in a systematic review of the literature, which tried to find out whether patients with osteoporosis had reduced BMD in the jawbones, it was concluded that because of the small number of studies and their heterogeneity, it was not possible to draw a reliable conclusion [22]. Therefore, more studies with larger sample sizes are needed to find out if and how osteoporosis affects the jawbones.

This study aimed to determine the impact of general bone mineral density (BMD) on the quality and quantity (the volume of the cortical and trabecular bone) of the edentulous mandible in postmenopausal women.

## 2. Materials and Methods

This cross-sectional study included 127 edentulous postmenopausal women aged 52 to 91 (mean age of 70.4 ± 8.9 years) who underwent a CBCT investigation for planning their dental implant treatment. The study included patients who attended Riga Stradins University Institute of Stomatology Clinic of Prosthodontics (Riga, Latvia) from October 2017 to November 2018.

### 2.1. Ethical Considerations and Study Registration

The study was conducted in accordance with the Declaration of Helsinki. Patients in the study were included with their approval, which was recorded in the agreement protocol. The study’s protocol was approved by the RSU Ethics committee (No. 28/05/10.2017). The study was registered in the ISRCTN registry (ID ISRCTN10873942). The STROBE guidelines were followed.

### 2.2. Inclusion and Exclusion Criteria

The study included female patients from the clinic of prosthetic dentistry at RSU’s Institute of Stomatology who had lost their teeth at least five years prior (as determined by interviewing patients and by clinical and radiological investigations); had been using conventional full dentures for at least 3 years, made at the same technical laboratory by using the same standards, and which had been provided with the same usage guidelines (determined according to the patients’ clinical records); and who had at least two years since the beginning of menopause (determined by patient self-declaration). Menopause was defined as when a woman had retrospectively experienced 12 months of amenorrhea without other obvious reasons (pathological or physiological).

Women with diseases and conditions that could cause secondary osteoporosis (diabetes, Cushing’s syndrome, hyperparathyroidism, thyrotoxicosis, kidney disease, rheumatoid arthritis, organ transplantation, etc.) were not included in the study. In addition, patients with early menopause (up to the age of 45) or surgery-induced menopause were excluded from the study. Patients who were taking medications affecting bone metabolism (bisphosphonates, glucocorticoids, strontium ranelate, calcitonin selective estrogen receptor modulators, HRT, active vitamin D metabolites, teriparatide, etc.), except for calcium taken at a dose of less than 1000 mg/day and vitamin D at a dose of less than 800 IU/day, either at the time of the study or one year before the start of the study, were also excluded from the study. Smokers and alcohol abusers (more than 14 alcohol units per week) were also not included in the study. Women with significant pathologies or inflammation in the jawbones were not included in the study.

Those who had no indication or had already had DXA examinations in the last year were also not included in the study.

### 2.3. Dual-Energy X-ray Absorptiometry

The BMD of the lumbar spine (L2–L4) and both femoral necks (total hip mean) was measured by dual-energy X-ray absorptiometry (DXA) (Lunar DEXA DPX-NT, GE Medical Systems). All investigations were performed by a single experienced specialist. The worst T-score reading (the number of standard deviations above or below the mean for a healthy 30-year-old adult of the same age, sex, and ethnicity) from both was considered. Patients were divided into three groups according to the DXA results: normal BMD (T-score ≥−1.0), osteopenia (T-score −1.0 to −2.5), and osteoporosis (T-score ≤ 2.5) [17]. Before the DXA investigation, each patient’s height and weight were measured. Body mass index (BMI) was calculated by dividing the patients’ mass in kilograms by the square height in meters (BMI = kg/m^2^).

### 2.4. CBCT Examinations

The study included patients who had undergone CBCT investigations at RSU’s Institute of Stomatology using the same equipment (i-CAT Next generation, KaVo Dental GmbH, Germany, Imaging Sciences International, Hatfield, PA, USA) and the same implant planning protocol, as follows: 120 kVp, 5 mA, an exposition time of 4 s, a voxel size of 0.3 mm, and a FOV size of 230 × 115 mm.

### 2.5. CBCT Measurements

**Figure 1:**
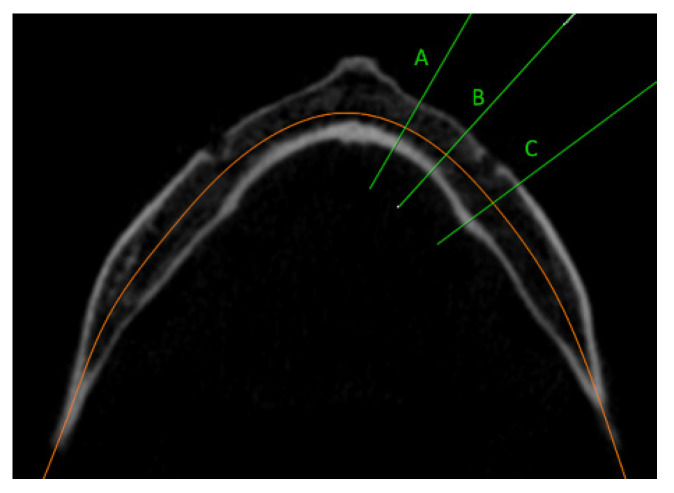
CBCT axial view of selected regions of the mandible: (A) lateral incisor region (9 mm from the midline of the mandible); (B) first premolar region (6 mm anteriorly from the middle of the mental foramen); (C) first molar region (6 mm distal from the middle of the mental foramen).

The acquired data were processed and analyzed using OnDemand3D^TM^ (Cybermed Inc., Seoul, Korea). In the dental module system, three regions of the mandible were selected in the axial view, namely the lateral incisor region (9 mm from the midline of the mandible), the first premolar region (6 mm anteriorly from the middle of the mental foramen), and the first molar region (6 mm distal from the middle of the mental foramen) (Figure 1).

**Figure 2:**
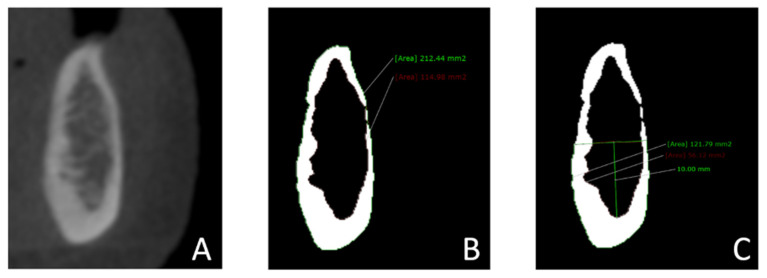
Measurements of the mandibular bone volume in cross-sectional images: (**A**) A cross-sectional slice was obtained in the regions of the lateral incisor, first premolar, and first molar. (**B**) Images were converted to binary images (black and white) to distinguish cortical bone from trabecular bone by adjusting the window’s level (960 gray values) and width (0 gray values). The total bone volume and trabecular bone volume in the complete mandible were measured. (**C**) The total bone volume and the trabecular bone volume in the basal part of the mandibular body were measured. The basal part of the mandibular body was defined as the entire mandibular area measuring 10 mm in height, starting at the most caudal point of the cortical–trabecular bone border and extending in the coronal direction.

A cross-sectional image was obtained for each of these areas. For each slice, the window level and width were adjusted to gray values of 960 and 0, respectively. In this way, the images were converted to binary images (black and white) to distinguish the cortical bone from the trabecular bone [28,29]. In each region (cross-sectional slice), the total mandibular volume and the trabecular bone volume were measured. The cortical bone volume was obtained by subtracting the trabecular bone volume from the size of the total bone volume. All measurements were also performed in the basal part of the mandibular body, with an area of 10 mm in height, starting at the most caudal point of the cortical–trabecular bone border and extending in the coronal direction [30] (Figure 2). If the bone’s height was under 10 mm, basal measurements were not performed and were not included. The cortical, trabecular, and total bone volume of the alveolar process of the mandible were calculated by subtracting the measurements of the basal part of the mandibular body from the measurements of the complete mandibular area.

All measurements were performed on the right side of the mandible; if this was not possible due to artifacts, the measurement was performed on the left side of the mandible.

**Figure 3:**
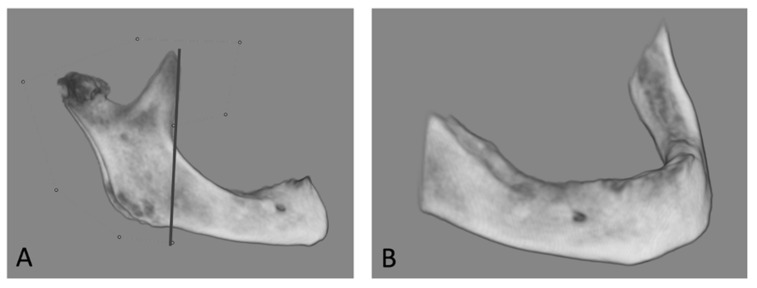
Measurements of the mandibular bone volume with Dolphin Imaging Plus^TM^. (**A**) The mandible was separated from the surrounding structures. A line perpendicular to the border of the mandible was drawn along the front of the coronoid process. (**B**) The total size (volume) of the mandible in front of the line was measured.

Dolphin Imaging Plus^TM^ (Dolphin 11.7, Dolphin Imaging and Management Solutions, Chatsworth, CA, USA) was used to separate the mandible from the surrounding structures. A line perpendicular to the border of the mandible was drawn along the front of the coronoid process. The total volume of the area ahead of the mandible was measured (Figure 3).

The CBCT images were analyzed using an LCD monitor with a resolution of 1920 × 1200 (one 24.1-inch LG monitor FlexScan S2202W; EIZO, Nano Corporation, Tokyo, Japan). The measurements were performed by one experienced observer with 15 years of experience in CBCT interpretation in general dental practice. To determine the repeatability of the measurements, all measurements were repeated at least 2 weeks apart.

### 2.6. Statistical Analysis

G* Power version 3.1.9.7 was used for calculating the sample size. The sample size was calculated on the basis of data obtained from a pilot study involving 37 edentulous patients [31]. The study’s power was assumed to be 80% (Type II errors), alpha ≤ 5% (Type I errors), and the effect size was 0.3, indicating that a total sample size of 105 participants would have adequate statistical power.

The data were analyzed using the statistical program R (studio version 3.6.3) [32]. Intra-examiner agreement was assessed by intra-class correlation. Descriptive statistics were calculated by grouping by diagnosis.

First, differences in bone measurements among the groups were evaluated using an ANOVA test with Bonferroni correction. Next, a linear regression model was created to adjust for the volume of the mandible, BMI, and the patient’s age, and to isolate the effect of the DXA diagnosis on the measurements. The significance value was set to 5%.

## 3. Results

According to the DXA results, 39 women had normal BMD, 57 women had osteopenia, and 31 had osteoporosis. There were statistically significant differences among the groups according to weight and BMI, but there was no statistically significant difference among the different BMD groups according to the women’s age or height (Table 1).

### 3.1. Cortical Bone

**Figure 4:**
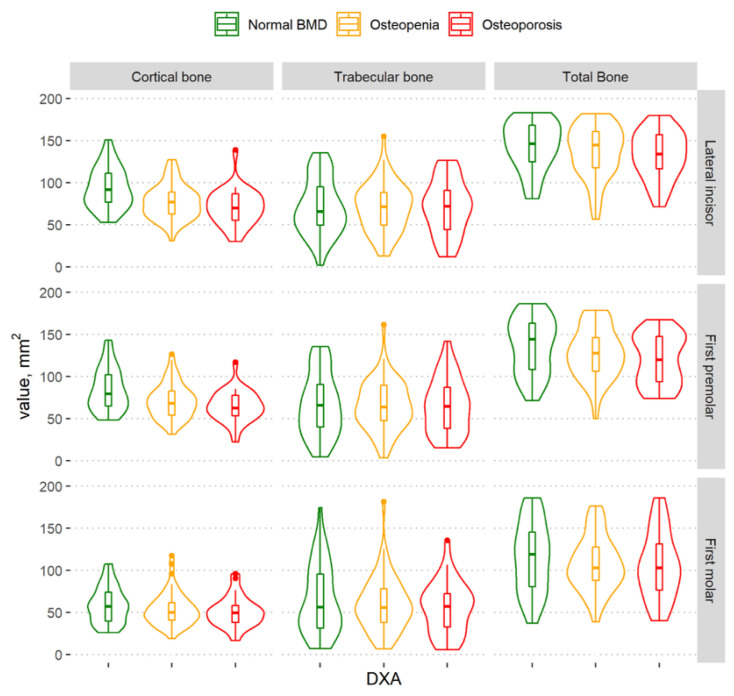
Violin plots of the cortical, trabecular, and total bone volume measurements in different BMD groups in the regions of the lateral incisor, first premolar, and first molar in the complete mandibular area.

**Figure 5:**
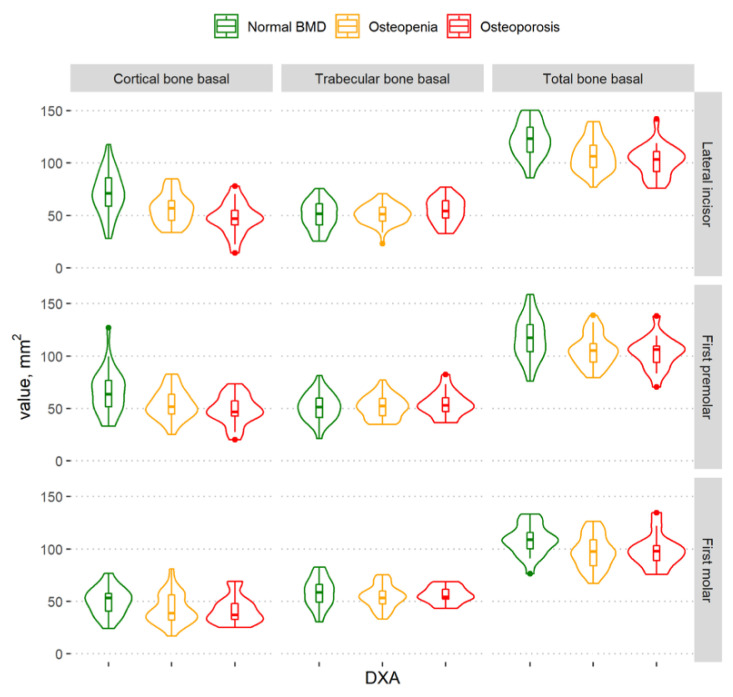
Violin plots of the cortical, trabecular, and total bone volume measurements in the different BMD groups in the regions of the lateral incisor, first premolar, and first molar in the basal part of the mandibular body.

The cortical bone volume decreased as BMD decreased (Figure 4 and Figure 5). A statistically significant difference among the different BMD groups according to the amount of cortical bone was observed in the lateral incisor and first premolar regions, both in the complete mandibular area and in the basal part of the mandibular body. However, there was no statistically significant difference among the different BMD groups according to the cortical bone in the alveolar part of the mandible (Table 2).

A more detailed analysis (ANOVA with Bonferroni correction) showed that the cortical bone volume differed statistically significantly between the normal BMD and osteoporosis groups (cortical bone in the lateral incisor region: complete mandibular area (*p* < 0.0001) and the basal part of the mandibular body (*p* ≤ 0.0001); cortical bone in the first premolar region: complete mandibular area (*p* = 0.001) and the basal part of the mandibular body (*p* = 0.02)) and between the normal BMD and osteopenia groups (cortical bone in the lateral incisor region: complete mandibular area (*p* = 0.001) and the basal part of the mandibular body (p ≤ 0.0001); cortical bone in the first premolar region: complete mandibular area (*p* = 0.008) and the basal part of the mandibular body (*p* = 0.01)). No statistically significant difference was observed between women with osteoporosis and those with osteopenia.

### 3.2. Trabecular Bone

There was no statistically significant difference between the different BMD groups according to the trabecular bone volume in different regions of the mandible, nor in the complete mandibular area, the basal part of the mandibular body, or in the alveolar part of the mandible (Table 2).

### 3.3. Total Mandibular Bone Volume

The graph shows that the total mandibular bone volume decreased with a decrease in BMD in all regions of the mandible (Figure 4); however, a statistically significant difference among the groups was found in the lateral incisor region only. There was no statistically significant difference among groups according to the total bone volume in the alveolar part of the mandible (Table 2).

In the basal part of the mandibular body (Figure 5), statistically significant differences among the different BMD groups were observed in all mandibular regions (Table 2). In a more detailed analysis, there was a statistically significant difference in the basal part of the mandibular body between the osteoporosis and normal BMD groups (lateral incisor region (*p* < 0.0001) and first premolar region (*p* = 0.02)) and between the normal BMD and osteopenia groups (lateral incisor region (*p* < 0.0001), first premolar region (*p* = 0.02), and first molar region (*p* = 0.04)). However, no statistically significant difference was observed between women with osteoporosis and those with osteopenia.

### 3.4. Regression Analysis

**Figure 6:**
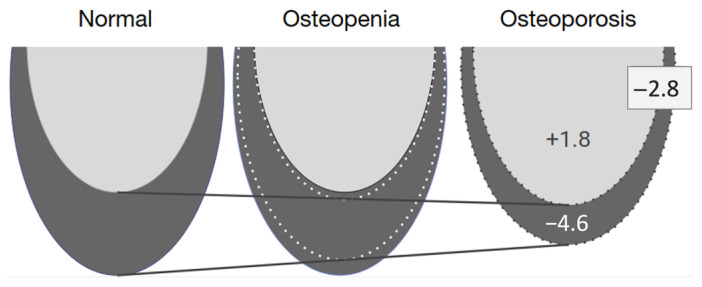
Summary of the adjusted regression model showing a +1.8 mm^2^ increase in the trabecular bone volume and a −4.6 mm^2^ decrease in the cortical bone volume, resulting in a net bone decrease of −2.8 mm^2^ in patients with osteoporosis. The trabecular bone is shown in light gray, and the cortical bone is in dark gray.

For this analysis, we used the worst DXA score from the lumbar spine and both femoral necks. Scatterplots with regression line were used to detect outliers. We decided to exclude five outliers, because these were patients with extremely resorbed edentulous jaws who had lost their teeth at a young age. This could have caused errors in the measurements, as such patients have a very small amount of trabecular bone. The regression model was adjusted for age, mandibular size (volume), and BMI. All bone areas (lateral incisors, first premolars, and first molars) were merged. A linear regression analysis revealed a similar trend to that found before, except that there was also a statistically significant increase in the trabecular bone volume when BMD decreased (Table 3). The regression model indicated that as BMD decreased and the patient progressed from a normal state to developing osteopenia and then osteoporosis, the mandibular bone changed with an increase in the proportion of trabecular bone volume and a decrease in the cortical bone volume, as shown in Figure 6.

### 3.5. Agreement or Repeatability of the Measurements

The intra-examiner observations demonstrated high repeatability for all measurements, with an intra-class correlation coefficient of >0.90 (varying from 0.90 to 0.99).

## 4. Discussion

RRR is affected by anatomical, metabolic, mechanical, and prosthetic factors [1]. Some researchers believe that metabolic factors, including osteoporosis, play a major role in RRR [2,14].

Studies on the effects of osteoporosis on RRR are contradictory. Some clinical trials have shown an association between the severity of RRR and general BMD [20,22,23], but others have not been shown such associations [24,25]. Few studies have examined alveolar bone resorption by determining the degree of RRR in all three dimensions [20,21,26]. The study groups were often relatively small [26] and not all studies used objective diagnostic methods for osteoporosis [20]. Measurements of BMD in the femoral neck and lumbar spine using the DXA method are considered the gold standard for diagnoses of reduced BMD [17].

Our previous study, in which the degree of RRR of the edentulous mandibles was determined by CBCT examinations, and the BMD of the lumbar spine and femoral neck was determined using DXA, failed to demonstrate that osteoporosis would affect RRR in edentulous mandibles [21]. As this study group was relatively small and the RRR of the edentulous jaws varied greatly between individuals, we decided to conduct the study by significantly increasing the study group. The results of the current study showed that the total cross-sectional volume of the mandibular bone was significantly smaller in the lateral incisor area in women with osteoporosis. However, after a regression analysis, in which the results were adjusted for age, BMI, and mandibular size (volume), we no longer observed a statistically significant relationship. The women’s age and BMI were included in the regression analysis because these are risk factors for osteoporosis [16]. Moreover, with increasing age, more pronounced RRR can be observed [33]. Further, the volume of the mandible was included to smooth out anatomical variations in the mandibular size across individuals.

Examining the effect of osteoporosis on the alveolar and basal part of the mandible in more detail revealed that the bone volume in the basal part of the jawbone was significantly smaller in all regions of the mandible in women with osteoporosis. In contrast, no statistically significant difference was observed among the different BMD groups according to the alveolar part of the mandible. Similar results were obtained in the regression analysis after adjusting for age, BMI, and jaw size. Our results are consistent with the view that osteoporotic changes can be better observed in the basal part of the mandible because it is relatively stable [34,35]. On the other hand, it is thought that the basal part of the mandible is less susceptible to jaw resorption [35]; however, our study showed that the basal part of the mandible was also negatively affected by osteoporosis.

This study failed to show that the alveolar part of the mandible was affected by osteoporosis. The results of this study are consistent with our previous study, which found no relationship between general BMD and the height and width of edentulous jaws in the alveolar bone of the mandible [21], and with other studies [24,25]. However, certain studies have found such a connection [20,22,23,26]. It is difficult to compare studies because different methods of determining bone resorption have been used, and only a few have used three-dimensional X-ray images [21,26]. The contradictory results and the fact that, in our study, the basal part of the mandible was significantly affected by osteoporosis but such an association was not observed in the alveolar part could be because osteoporosis is most likely to affect the whole mandible to some extent, but other factors have a more significant effect on the resorption of the alveolar process. In our study, some of these factors affecting RRR were considered. To exclude the effect of the insufficient size of the denture base and inadequate occlusion on the RRR [36], in our study, all complete dentures were made in one clinic according to the same criteria and had an adequate denture base and occlusion. Patients were included in the study if the last teeth had been extracted at least five or more years ago, and the prostheses had been used by the women for at least three years or more, as faster RRR was observed in the first years after tooth extraction, with 23.09% bone loss [37]. This more rapid bone loss is influenced by local rather than systemic factors [38]. All patients also received the same instructions for using dentures (not to use dentures at night) so that the effects of complete dentures on RRR would be as similar as possible for all patients.

Among the shortcomings of the study, it was not possible to control all the factors that could affect the RRR; for example, the exact time of extraction of the last teeth, the reasons for the extraction (periodontal disease, dental caries, etc.), and whether the previously made prostheses were adequate remained unknown. Most likely, all of these and possibly other factors play an important role in bone resorption in the alveolar part of the mandible. Our study showed that osteoporosis only affects the basal part of the mandible, which is apparently less affected by the factors mentioned above.

In addition to the amount of bone, the quality of the bone is important when inserting dental implants [4,5]. The term “bone quality” is not clearly defined in the literature. It includes physiological and structural aspects and the degree of bone mineralization [39].

In a systematic review of the literature, which included five studies, four found a correlation between the jawbone’s BMD and overall BMD in patients with osteoporosis. Because of the small number of studies included and their heterogeneity, the authors could not draw a reliable conclusion [27].

In implantology, the classification proposed by Lekholm and Zarb, [4] based on the mass of cortical and trabecular bone and their ratio, is often used. The amount of cortical bone is an important factors used to characterize bone quality [4]. This is especially important for ensuring the stability of the primary implant [10,11].

The results of our study showed that the cortical bone was thinner in women with reduced BMD in the lateral incisors and first premolar regions. Analyzing this in more detail revealed that such an association was particularly pronounced in the basal part of the mandible, but not in the alveolar part of the mandible. These results could be explained by the fact that the cortical bone in the alveolar part of the mandible is anatomically thinner [29,30] and more prone to resorption [28]. A similar study to ours was conducted by Naitoh et al. [28], in which the thickness of the cortical bone was measured in four different areas of the mandible, and the BMD was determined in the lumbar spine by DXA. In contrast to our study, a weak statistically significant correlation was found between the amount of cortical bone and BMD in the lumbar spine in only one selected area of the mandible. These differences in the studies’ results may have occurred because the earlier study used a relatively small group of patients and a different method to estimate the cortical bone volume [28].

A study by Munakata [13] and colleagues found that menopause influences the decrease in cortical bone and the increase in the trabecular bone volume of the mandible in the molar region in women. Although menopause is often associated with osteoporosis caused by decreased estrogen levels, no diagnosis of osteoporosis was carried out in their study. Another study also found that women have less cortical bone in the molar region than men, and that this is also decreased with increased age in women [40]. A study in Austria showed that cortical bone is not affected by jawbone resorption and that women lose more bone than men do, which could be explained by hormonal differences and osteoporosis. However, they did not have information on whether the patients had osteoporosis or other metabolic diseases [30]. In another study, the results showed that cortical bone was not affected by jaw resorption [41].

Although these studies’ goals and methodologies were different, the results of our study are consistent with those of studies that show that the mandibular cortical layer becomes thinner and changes in structure in patients with reduced BMD, and that it can be used to determine the risk of osteoporosis [42,43].

A regression analysis, in which the results were adjusted for age, BMI, and mandibular volume, showed that women with osteoporosis had a smaller total bone volume in the basal part of the mandible and a smaller cortical bone volume, but that the trabecular bone increased slightly with an increase in BMD, but not to a similar extent to the loss of cortical bone. This makes it possible to hypothesize that the total bone volume is lost at the expense of cortical bone. In addition, the cortical bone also shrinks from the side of the trabecular bone. This means that the cortical bone is lost at both the outer and inner edges of the bone (Figure 6). Similar skeletal changes are seen in postmenopausal women with osteoporosis who have a thinner and more porous cortical bone in the distal radius and tibia than in women with a normal BMD [44]. Changes in the thickness and geometry of the cortical bone are significant risk factors for bone fractures [45]. Similarly, some researchers have stated that mandibular stability depends more on cortical bone than on trabecular bone [46].

A more pronounced effect of osteoporosis in our study was observed in the frontal part of the mandible. This could be explained by anatomical features. Although the proportion of cortical bone in the basal part of the mandible did not change with the degree of RRR, the cortical bone was located more in the distolingual part of the severely resorbed mandible [30].

The strengths of the study are that the study did not include patients with systemic diseases that may have affected BMD, or patients taking medication to treat osteoporosis or reduce general BMD. Patients who smoked were also excluded.

On the other hand, the fact that patients with a relatively wide age range were included can be considered as a weakness of the study. There is a belief that osteoporosis must be present for a long enough time to affect the jawbones [14]. Therefore, the youngest patients in this study may not have had enough time for osteoporosis to have affected the edentulous mandible.

Nevertheless, the results of this study provide important information to clinicians on how osteoporosis affects the edentulous mandible; however, further research on the effects of osteoporosis on the maxilla is warranted.

## 5. Conclusions

In conclusion, postmenopausal women with osteoporosis have lower total and cortical bone volume in the basal part of the edentulous mandible than women with a normal BMD. Osteoporosis affects the quantity and quality of bone in the basal part of the edentulous mandible.

## Figures and Tables

**Table 1:** Means, standard deviations, and one-way ANOVA results for the demographic data of the normal BMD, osteopenia, and osteoporosis groups.

Factor	Overall Mean (SD) (n = 127)	Normal BMD Mean (SD) (n = 39)	Osteopenia Mean (SD) (n = 57)	Osteoporosis Mean (SD) (n = 31)	*p*-Value
Age	70.4 (8.9)	68.9 (8.9)	70.4 (9.1)	72.5 (8.2)	0.24
Height	160.0 (5.9)	160.9 (5.4)	159.3 (6.1)	159.9 (6.0)	0.43
Weight	73.1 (15.7)	82.2 (16.7)	71.7 (12.6)	64.2 (13.8)	<0.001 *
BMI	28.6 (5.9)	31.8 (6.0)	28.3 (5.2)	24.9 (4.6)	<0.001 *

SD, standard deviation; BMI, body mass index; BMD, bone mineral density. The *p*-values were from one-way ANOVA, * *p* ≤ 0.05.

**Table 2:** Means, standard deviations, and one-way ANOVA results for total bone volume, trabecular bone volume, and cortical bone volume in the complete, alveolar part, and basal part of the mandibular body in the normal BMD, osteopenia, and osteoporosis groups.

		Normal BMD	Osteopenia	Osteoporosis	
Region		Mean (SD) Number	Mean (SD) Number	Mean (SD) Number	*p*-Value
**Lateral incisor**					
	Cortical bone, CM	93.7 (24.1) n = 39	77.2 (20.5) n = 57	70.7 (21.4) n = 30	<0.001 *
	Cortical bone, basal	71.3 (20.6) n = 31	56.3 (14.0) n = 49	47.5 (15.0) n = 23	<0.001 *
	Cortical bone, alveolar	27.5 (10.0) n = 31	22.8 (10.3) n = 49	22.9 (12.4) n = 23	0.12
	Trabecular bone, CM	70.7 (33.0) n = 39	70.9 (28.9) n = 57	70.9 (32.4) n = 30	>0.99
	Trabecular bone, basal	50.4 (13.3) n = 31	51.1 (9.3) n = 49	54.7 (12.1) n = 23	0.35
	Trabecular bone, alveolar	29.19 (18.6) n = 31	26.6 (19.5) n = 49	28.9 (19.9) n = 23	0.82
	Total bone, CM	164.4 (41.2) n = 39	148.1 (38.6) n = 57	141.5 (38.5) n = 30	0.04 *
	Total bone, basal	121.7 (17.0) n = 31	107.4 (15.4) n = 49	102.1 (14.9) n = 23	<0.001 *
	Total bone, alveolar	56.7 (25.3) n = 31	49.5 (26.6) n = 49	51.8 (28.8) n = 23	0.50
**First premolar**					
	Cortical bone, CM	84.4 (25.2) n = 39	70.8 (20.2) n = 57	66.7 (18.2) n = 30	<0.001 *
	Cortical bone, basal	65.5 (21.6) n = 29	53.8 (13.8) n = 45	48.6 (13.8) n = 19	0.001 *
	Cortical bone, alveolar	20.5 (12.1) n = 29	17.7 (10.8) n = 45	17.6 (11.5) n = 19	0.54
	Trabecular bone, CM	66.5 (36.9) n = 39	65.4 (29.3) n = 57	64.9 (32.7) n = 30	0.98
	Trabecular bone, basal	51.5 (13.2) n = 29	51.6 (10.7) n = 45	54.2 (12.0) n = 19	0.69
	Trabecular bone, alveolar	29.6 (23.2) n = 29	23.5 (18.5) n = 45	27.5 (19.3) n = 19	0.43
	Total bone, CM	150.9 (43.7) n = 39	136.2 (38.7) n = 57	129.5 (40.4) n = 30	0.08
	Total bone, basal	117.0 (20.3) n = 29	105.5 (14.7) n = 45	102.8 (15.3) n = 19	0.005 *
	Total bone, alveolar	50.1 (32.1) n = 29	41.2 (26.9) n = 45	45.0 (28.9) n = 19	0.44
**First molar**					
	Cortical bone, CM	58.9 (21.6) n = 39	53.8 (19.4) n = 57	50.8 (17.8) n = 31	0.21
	Cortical bone, basal	50.0 (13.3) n = 19	42.3 (14.3) n = 30	41.9 (13.3) n = 15	0.14
	Cortical bone, alveolar	15.0 (10.3) n = 19	14.5 (8.6) n = 30	16.2 (8.3) n = 15	0.85
	Trabecular bone, CM	64.7 (41.3) n = 39	59.8 (31.9) n = 573	55.4 (30.9) n = 31	0.54
	Trabecular bone, basal	58.4 (14.0) n = 19	54.5 (10.8) n = 30	56.2 (7.8) n = 15	0.51
	Trabecular bone, alveolar	35.9 (26.1) n = 19	25.8 (21.7) n = 30	20.5 (19.8) n = 15	0.13
	Total bone, CM	123.5 (49.5) n = 39	113.5 (40.1) n = 57	106.2 (39.8) n = 31	0.24
	Total bone, basal	108.4 (14.5) n = 19	97.1 (15.6) n = 30	98.2 (15.6) n = 15	0.04 *
	Total bone, alveolar	50.6 (31.0) n = 19	40.3 (27.6) n = 30	36.7 (21.9) n = 15	0.29

*p*-value from one-way ANOVA; * *p* ≤ 0.05. CM, bone in the complete mandible area; SD, standard deviation; BMD, bone mineral density.

**Table 3:** Linear regression analysis to detect connections among the cortical, trabecular, and total bone volume in the complete mandibular area and the basal part of the mandibular body with the worst DXA T-scores after excluding outliers.

Model	Factors	Est. Coeffic.	S.E.	*p*-Value	*R^2^*
Cortical bone, CM	Intercept	109.15	11.66	<0.001	
	DXA	3.93	0.94	<0.001	
	Age	−0.76	0.12	<0.001	
	BMI	−0.94	0.2	<0.001	
	Mandibular volume	2.39	0.27	<0.001	
					0.34
Cortical bone, basal	Intercept	111.82	11.53	<0.001	
	DXA	4.64	0.85	<0.001	
	Age	−0.78	0.11	<0.001	
	BMI	−0.19	0.18	0.3	
	Mandibular volume	0.52	0.26	0.05	
					0.32
Cortical bone, alveolar	Intercept	17.0	7.72	0.03	
	DXA	0.4	0.57	0.48	
	Age	−0.1	0.07	0.17	
	BMI	−0.42	0.12	<0.001	
	Mandibular volume	1.1	0.17	<0.001	
					0.21
Trabecular bone, CM	Intercept	−96.46	14.57	<0.001	
	DXA	−4.54	1.17	<0.001	
	Age	0.73	0.15	<0.001	
	BMI	0.24	0.25	0.32	
	Mandibular volume	5.2	0.33	<0.001	
					0.45
Trabecular bone, basal	Intercept	−9.3	7.92	0.24	
	DXA	−1.83	0.59	<0.001	
	Age	0.46	0.07	<0.001	
	BMI	0.15	0.13	0.23	
	Mandibular volume	1.05	0.18	<0.001	
					0.22
Trabecular bone, alveolar	Intercept	−50.43	13.69	<0.001	
	DXA	−1.95	1.01	0.05	
	Age	0.2	0.13	0.12	
	BMI	0.18	0.22	0.42	
	Mandibular volume	2.73	0.3	<0.001	
					0.26
Total bone, CM	Intercept	29.22	16.46	0.08	
	DXA	−0.36	1.34	0.79	
	Age	0.05	0.17	0.75	
	BMI	−0.96	0.28	<0.001	
	Mandibular volume	6.41	0.43	<0.001	
					0.43
Total bone, basal	Intercept	102.51	11.41	<0.001	
	DXA	2.8	0.84	<0.001	
	Age	−0.32	0.1	<0.001	
	BMI	−0.04	0.18	0.83	
	Mandibular volume	1.56	0.25	<0.001	
					0.29
Total bone, alveolar	Intercept	4.54	15.93	0.78	
	DXA	−1.2	1.16	0.3	
	Age	0.18	0.14	0.2	
	BMI	−0.96	0.25	<0.001	
	Mandibular volume	2.26	0.39	<0.001	
					0.18

S.E., standard error; DXA, worst DXA T-score; CM, bone volume in the complete mandible area; BMI, Body mass index.

## Data Availability

Data supporting the conclusions of this study are available upon request from the corresponding author (A. Slaidina).
